# Melanoma tumor growth is accelerated in a mouse model of sickle cell disease

**DOI:** 10.1186/s40164-015-0014-1

**Published:** 2015-07-07

**Authors:** Jintao Wang, Jennifer Tran, Hui Wang, Wei Luo, Chiao Guo, David Harro, Andrew D. Campbell, Daniel T. Eitzman

**Affiliations:** Department of Internal Medicine, Cardiovascular Research Center, University of Michigan, 7301A MSRB III, 1150 West Medical Center Drive, Ann Arbor, MI 48109-0644 USA; Department of Pediatrics, University of Michigan, Ann Arbor, MI USA; Chemical Pathology, University of Michigan Hospital, Ann Arbor, MI USA

**Keywords:** Sickle cell disease, Melanoma, Angiogenesis, Heme oxygenase-1

## Abstract

**Background:**

The effect of sickle cell disease (SCD) on tumor growth is unknown. Sickled red blood cells may form aggregates within the microvasculature of hypoxic tumors and reduce blood flow leading to impairment of tumor growth. However, there is a paucity of data related to tumor growth in SCD.

**Methods:**

To investigate the effect of SCD on tumor growth in a melanoma model, we generated SCD and control mice using bone marrow transplantation and inoculated the chest wall with B16-F10 melanoma cells. Tumor growth was monitored and angiogenesis was studied in vivo and in vitro.

**Results:**

From day 1 to 21, tumor growth rate was nearly identical between SCD and WT mice, however from day 22 to day 29 tumor growth was accelerated in SCD mice compared to WT mice. Disparity in tumor size was confirmed at autopsy with an approximate 2-fold increase in tumor weights from SCD mice. Tumors from SCD mice showed increased vascularity and elevated levels of heme oxygenase-1 (HO-1). HO-1 inhibition with zinc protoporphyrin (ZnPP) blocked the angiogenic and tumor growth response to SCD in vivo and the response to hemin in vitro.

**Conclusions:**

Growth of melanoma tumors is potentiated in a mouse model of SCD. Therapies targeting angiogenesis or HO-1 may be useful in SCD patients with malignant tumors.

## Background

Malignant tumors are characterized by regions of hypoxia as the growth and metabolism of the tumor outpaces the supply of oxygenated blood [1]. These areas of hypoxia have been shown to induce local sickling of infused red blood cells that carry the mutation for sickle cell disease (SCD) [2]. Since SCD is associated with elevated heme oxygenase-1 (HO-1) due to chronic hemolysis [3], SCD may promote tumor growth. HO-1 overexpression has been previously shown to promote the growth of some tumors, including melanoma [4]. SCD has also been shown to promote angiogenic responses [5], which could promote tumor growth [6].

To our knowledge, the effect of host SCD on tumor growth has not been previously reported. Because of the potential of SCD to modify the growth of malignant tumors, we tested the growth rate of murine melanoma tumors in a mouse model of SCD.

## Materials and methods

### Ethics statement

All procedures complied with the Principles of Laboratory and Animal Care established by the National Society for Medical Research and were approved by the University of Michigan Committee on Use and Care of Animals.

### Mice

Wild-type C57BL6/J male mice were purchased from the Jackson Laboratory (Bar Harbor, ME). Male 8 week old mice were fed a standard laboratory rodent diet (#5001, TestDiet, Richmond, Ind) in specific pathogen-free facilities. Donor mice carrying the homozygous sickle cell mutation (Hbb^hβs^/^hβs^) were originally from University of Alabama at Birmingham [7] and these mice have subsequently been bred to C57BL6/J mice to generate heterozygous mice, which were then intercrossed to produce the homozygous Hbb^hβs^/^hβs^ donors [8]. SCD and control experimental mice were then generated by bone marrow transplantation (BMT) from Hbb^hβs^/^hβs^ mice or wild-type (Hbb^+/+^) donors to wild-type C57BL6/J male recipients. All procedures complied with the Principles of Laboratory and Animal Care established by the National Society for Medical Research and were approved by the University of Michigan Committee on Use and Care of Animals.

### Cell culture

Murine melanoma (B16-F10, CRL-6475™, ATCC) and brain endothelial cells (bEnd.3, CRL-2299™, ATCC) were grown in Dulbecco’s modified Eagle medium (DMEM, Gibco Inc.) containing 10 % fetal bovine serum (FBS, Gibco Inc.) and passaged 2 to 3 times before use in assays.

### Bone marrow transplantation

Male donor mice for BMT were euthanized at 8–10 weeks of age and bone marrow was then flushed from femurs and tibias. Recipient mice were irradiated with 650 rads x 2 separated by a 3 hour interval (total of 1300 rads). Each recipient mouse was administered a 200 μl bone marrow suspension in PBS (2 × 10^7^cells/ml) via tail vein injection. Acid water (6 mM HCL, pH = 2.5) was provided to animals beginning 4 days before BMT to 4 weeks following BMT. Recipient mice were housed in a specific pathogen free animal facility.

### Tumor model

Melanoma tumors were induced by subcutaneous injection of 1 × 10^5^ B16-F10 murine melanoma cells over the left lateral chest wall. This melanoma cell line is from the same strain background as the recipient mice, thus it is not rejected. Tumor size and body weight were measured daily for four weeks. Tumor volumes were calculated using the formula 0.5 x length x width^2^ [9].

### HO-1 activity assay

The enzyme activity of HO-1 was measured as previously described [3]. Briefly, 100 mg frozen tumor tissue was homogenized in 250 μl PBS, and centrifuged at 18,000 g for 10 min at 4 ° C. The source of biliverdin reductase for each assay was prepared by centrifuging 2 mg WT homogenized liver. To initiate the reaction, 200 μl supernatant of tumor sample was added to a reaction system containing 0.8 mmol/L NADPH, 2 mmol/L glucose-6-phosphate, 0.2 units glucose-6-phosphate dehydrogenase, 0.2 mmol/L MgCl_2_, 0.02 mmol/L hemin, and 100 μl liver cytosol in a final volume of 300 μl. The reaction mixture was incubated for 60 min at 37 ° C in the dark and then stopped by mixing 1:1 with chloroform. The extracted bilirubin in the chloroform was measured at 464 nm subtracted by absorption at 530 nm. The HO activity was expressed as formation of bilirubin (pmol) per milligram of sample in 1 h. Units of activity are therefore pmol/mg/hr.

### HO-1 inhibition

The HO-1 inhibitor, zinc protoporphyrin IX (ZnPP, Sigma, St. Louis, MO, USA), was prepared in 0.1 N NaOH, and then titrated to pH 7.4 using 0.1 N HCl. ZnPP (2.5 mg/kg in 0.9 % NaCl) was given IP twice a week to tumor- bearing mice. For aortic ring assays, ZnPP was added at 2.5 μM.

### Tumor angiogenesis

At the end of the protocol, mice were euthanized and perfused with PBS. Tumors were fixed with formalin, paraffin-embedded and sectioned (5μm). Endothelial cells were stained with an anti-mouse CD31 monoclonal antibody (1:50, Abcam, Cambridge, MA). For quantitation of vessel density, the areas of highest neovascularization, “hot spots”, were identified by scanning the sections at low power (200X) followed by vessel counts at high power (400X), as previously described [10]. For each tumor section, five hot spots were identified and vessels were counted.

### RT-PCR

For HO-1 expression analyses, bEnd.3 cells were seeded in 6-well plates (Cat #3516, Corning Inc.) and allowed to form a confluent monolayer. DMSO or hemin at varying concentrations (three wells per concentration) were added to the medium for 5 hours before cells were harvested. RNA was isolated using a QIAGEN RNeasy Mini Kit (QIAGEN Inc., Valencia, CA) according to manufacturer’s instructions. Primer sets for specific amplification of murine HO-1 and glyceraldehyde-3-phosphate dehydrogenase (GAPDH) were purchased from Applied Biosystems (Carlsbad, CA). RT-PCR was performed using an ABI Prism 7000 Sequence Detection System (Applied Biosystems), with 200 ng RNA and 1 μL primer used per reaction. Results were analyzed using 7000 System SDS Software and the 2-ΔΔCT method [11]. HO-1 expression levels were presented as percentage of DMSO control.

### Aortic ring angiogenesis assay

For analysis of aortic ring angiogenesis, 150 μl Cultrex BME (R&D Systems, 3432-005-01) was added to each well of a 48-well plate and placed in an incubator for 30 minutes to solidify the matrix. WT BMT mice were euthanized, thoracotomy was performed and thoracic aorta was removed. Aortas were cleaned of adventitial tissue and blood and then serially cross-sectioned into 1–2 mm rings. For each group, four aortic rings were embedded in 300 μl Cultrex BME (R&D Systems, 3432-005-01) in plate wells. Complete culture medium with DMSO control or hemin (with or without ZnPP) was added and the rings were cultured for 7 days with 2 media changes. Images were taken and the number of sprouts was counted [12].

### Matrigel plug assay

For matrigel experiments, growth factor-reduced matrigel (300 μl; 3432-005-01, BD Bioscience) was injected subcutaneously into the ventral aspect of each mouse. WT mice treated with PBS (*n* = 3) or ZnPP (*n* = 5) and SCD mice treated with PBS (*n* = 5) or ZnPP (*n* = 5) were studied. 10 days after implantation, mice were euthanized and plugs were removed, fixed with formalin, embedded with paraffin, sectioned, and stained with anti-CD31 antibody. The vascular density was determined by counting number of blood vessels per matrigel plug section [13].

### Statistical analysis

Values are expressed as mean ± SD. The statistical significance of differences between two groups was determined by the student 2-tailed *t* test. For multiple comparisons, results were analyzed using two-way ANOVA, followed by Bonferroni post-test analysis. *P* < 0.05 was considered significant. For analysis of mice with metastasis, Fisher’s exact test was used. Pearson correlation test was performed for correlation analyses.

## Results

### Effect of SCD on melanoma growth rate

**Fig. 1:**
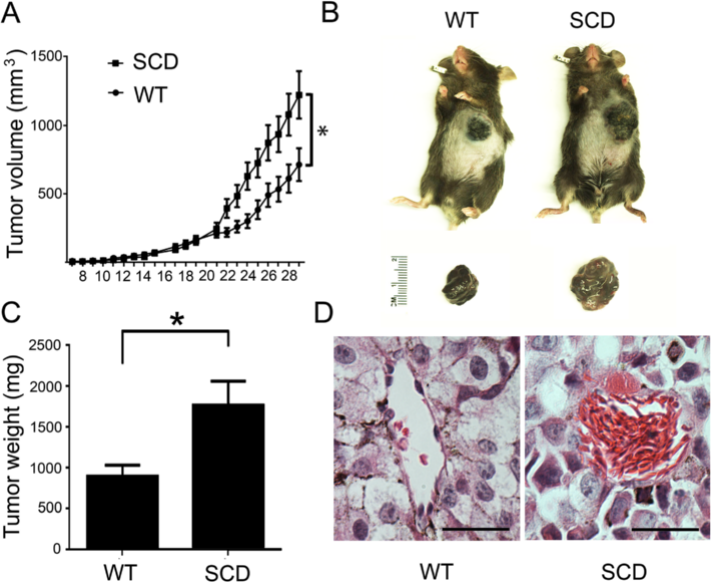
Accelerated tumor growth in SCD mice. **a** Growth curves of WT mice (*n* = 10) and SCD mice (*n* = 10). **b** Representative image of tumor bearing SCD and WT mouse at time of sacrifice. **c** Tumor weights of WT and SCD mice at time of sacrifice. **d** Sickled erythrocytes within tumor vessels from SCD mice. Scale bar = 25 μm

To determine the effect of SCD on the growth of malignant tumors, we implanted murine melanoma cells in SCD and WT recipients. Tumors were implanted over the left lateral chest wall so that they could be easily monitored and measured daily. Growth of tumors during the first 3 weeks was similar between the WT and SCD mice (Fig. 1a). From day 21 to 29, there was acceleration of tumor growth rate in both groups but the rate of growth was markedly increased in the SCD group compared to the WT group (Fig. 1a). Tumor volumes and weights were both increased in the SCD compared to WT mice at time of sacrifice (Fig. 1a, b, c). Body weights of mice at the beginning and end of the protocol were similar between the WT and SCD mice (24.93 ± 1.13 vs. 24.03 ± 0.79 grams on day 1; 25.46 ± 0.79 vs 25.74 ± 1.22 grams on day 29). Histological analyses showed frequent microvascular occlusions in tumors from SCD mice (Fig. 1d). Visible metastatic lung surface nodules were present in 40 % of wild-type mice and 55 % of SCD mice at the time of sacrifice. However, there was no significant difference between the two groups (p value for Fisher’s exact test = 0.67).

**Fig. 2:**
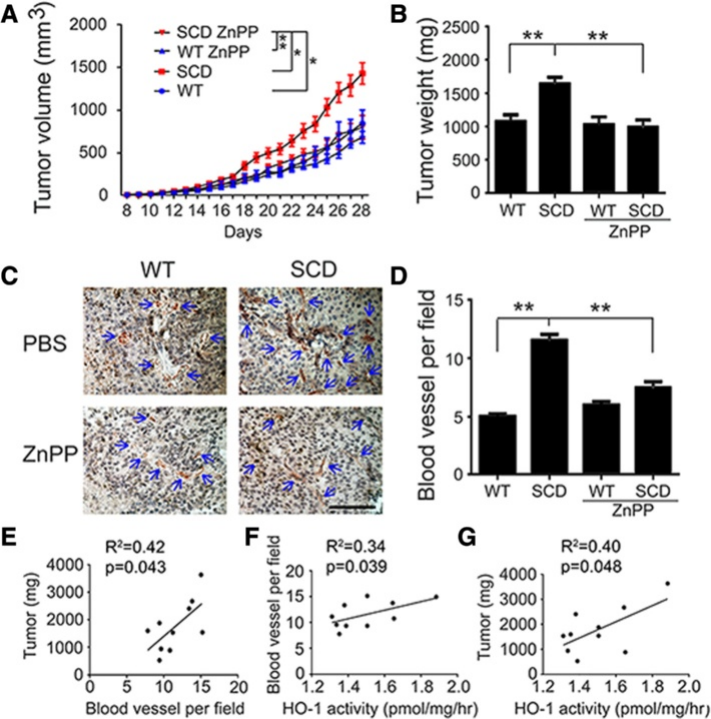
Accelerated tumor growth in SCD mice is prevented with suppression of HO-1. **a** Growth curves of WT mice (*n* = 10), SCD mice (*n* = 11), WT mice treated with ZnPP (*n* = 8) and SCD mice treated with ZnPP (*n* = 6). **b** Tumor weights of WT mice (*n* = 10), SCD mice (*n* = 11), WT mice treated with ZnPP (*n* = 8) and SCD mice treated with ZnPP (*n* = 6). **c** Representative image of blood vessel (CD31) staining in tumor hot spots from the 4 groups of mice, **d** Quantification of blood vessel density of tumors from the 4 groups of mice. Scale bar =100 μm. **e** Correlation of tumor weight and blood vessel density in SCD mice. **f** Correlation of blood vessel density and HO-1 activity in SCD mice. **g** Correlation of tumor weight and HO-1 activity in SCD mice

Elevated HO-1 expression has been shown to promote the growth of melanoma [4]. Since HO-1 has also been shown to be elevated in mice and human with SCD [3, 14, 15], we analyzed HO-1 activity in melanoma tumor tissue taken from SCD and WT mice at the time of sacrifice. HO-1 activity levels were higher in melanoma tumors from SCD mice compared to WT mice (1.12 ± 0.13 vs. 1.0 ± 0.08, respectively, *p* < 0.05). To determine the causal role of HO-1 in promoting tumor growth in mice with SCD, the tumor implantation experiments were repeated with the addition of the HO-1 inhibitor ZnPP. ZnPP (2.5 mg/kg) or vehicle control was administered IP twice per week to WT and SCD mice following tumor implantation. Consistent with the initial study, tumor growth rate was accelerated in SCD mice treated with only vehicle control, however, tumor growth rates in SCD mice treated with ZnPP were similar to WT mice. ZnPP had no effect on growth rate of tumors in WT mice (Fig. 2a). Tumor weights at sacrifice confirmed the suppression of tumor growth in SCD mice by ZnPP (Fig. 2b). To compare the HO-1 activity, the activity of WT mice treated with vehicle is set as 1 and the HO-1 activity in other groups was expressed as ratio to control. HO-1 activity was elevated in SCD mice treated with vehicle compared to WT mice treated with vehicle (1.3 ± 0.25 vs. 1.0 ± 0.11, *p* < 0.05), however, HO-1 activity was reduced in SCD mice treated with ZnPP (0.65 ± 0.10 compared to WT mice treated with vehicle, *p* <0.001 for comparison of SCD mice treated with vehicle compared to SCD mice treated with ZnPP). HO-1 activity was also reduced in WT mice treated with ZnPP (0.8 ± 0.11, p = 0.02).

### Angiogenic responses in SCD mice

The pattern of late accelerated melanoma growth in SCD mice suggested that enhanced tumor angiogenesis in SCD mice may support accelerated tumor growth [16]. Blood vessels in tumors were therefore quantitated as previously described, [10] and found to be more frequent in SCD tumors compared to WT tumors (Fig. 2c and d). Increased vessel density was not observed in SCD mice when mice were treated with ZnPP, indicating HO-1 may be one of factors promoting angiogenesis in SCD melanoma tumors (Fig. 2c and d). Correlation analysis between tumor weight, HO-1 activity, and vessel quantitation in the individual mice revealed that tumor size, HO-1 activity and vessel density are correlated in SCD mice (Fig. 2e, f and g). However, tumor weight was not correlated with HO-1 activity (R^2^ = 0.006, p = 0.84) in WT mice.

**Fig. 3:**
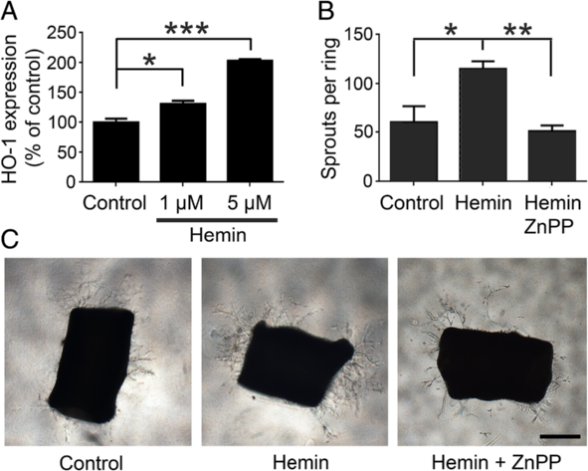
Hemin induces HO-1 expression in cultured endothelial cells and promotes angiogenesis from aortic rings. **a** HO-1 expression level in bEend.3 cells treated with DMSO (control), 1 μM hemin or 5 μM hemin. **b** Quantification of sprouts from aortic rings treated with DMSO (control), 1 μM hemin or 1 μM hemin with 2.5 μM ZnPP. **c** Representative images of sprouts from aortic rings treated with DMSO, 1 μM hemin or 1 μM hemin with 2.5 μM ZnPP. Scale bar = 500 μm

Hemin is elevated in SCD and has been shown to induce HO-1 [17]. To determine the effect of hemin on HO-1 induction in endothelial cells, hemin was incubated with endothelial cells in culture. Hemin induced a dose dependent increase of HO-1 expression in endothelial cells (Fig. 3a). To determine the angiogenic response to hemin, hemin was incubated with isolated aortic rings using an established aortic ring angiogenesis model [12]. Hemin increased the number of angiogenic sprouts (Fig. 3b and c). This effect was blocked with addition of ZnPP suggesting that hemin induced angiogenesis via HO-1.

**Fig. 4:**
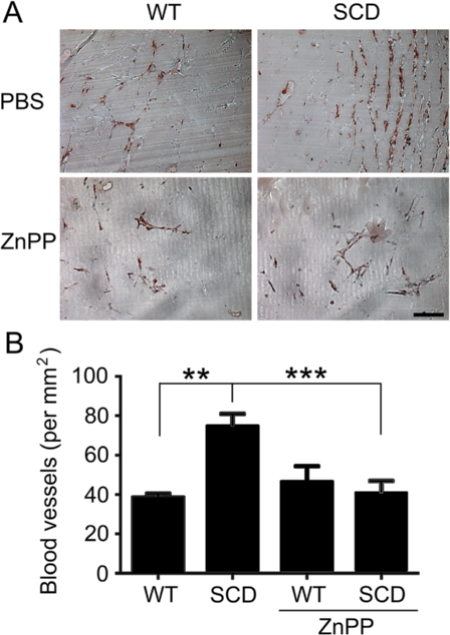
SCD promotes angiogenesis in matrigel implants which is suppressed via HO-1 suppression with ZnPP. **a** Representative images of CD31 staining in matrigel plug sections. **b** Quantification of vessels in matrigel plugs. Scale bar =100 μm

To next determine the host angiogenic response under endogenous conditions, matrigel was implanted in SCD and WT mice. Seven days following matrigel implantation, vessels were quantified and found to be increased in SCD compared to WT mice (Fig. 4a and b). The increased angiogenic response was blocked with ZnPP treatment (Fig. 4a and b).

## Discussion

The incidence, prevalence and outcomes of cancers in patients with sickle cell disease remains ill-defined [18]. Many factors associated with SCD in humans may affect the incidence and growth rates of cancer, including hydroxyurea treatment, chronic inflammation, transfusion-related infections, focal ischemia due to sickling, and chronic hemolysis. These factors may promote or impair the growth of tumors. To our knowledge, the effect of tumor growth in a host with SCD has not been described.

Murine models of SCD are available that mimic many of the abnormalities observed in humans with SCD [19–21]. Because of the limited fertility of SCD mice and the complexity involved in breeding heterozgyotes with background strain heterogeneity, bone marrow transplantation was used. Bone marrow transplantation from a donor sickle cell mouse to wild-type recipient can be used to generate large numbers of age and sex-matched SCD mice on a relatively homogenous genetic background. We have previously used this method to study the effects of SCD on vascular endpoints [3, 22]. The effects of SCD on the vasculature are complex leading to both adverse and protective responses depending on the vascular bed and insult [3, 22]. Since tumor growth may be regulated by angiogenesis [16], SCD could have major effects on tumor growth since SCD may represent a proangiogenic state [5]. Consistently, a previous study demonstrated that injection of sickle cell red blood cells accelerated 4T1 tumor growth in a murine model, an effect reversible with ZnPP treatment [2].

The murine B16-F10 melanoma model has been extensively studied in mouse models [23] and tumor growth has been shown to be affected by interventions that impact angiogenesis [24]. Because of the relatively rapid growth characteristics of the tumor, we tested the effect of SCD on melanoma tumor growth. Growth rates of the primary tumor were similar between SCD and WT mice during the early growth phase following subcutaneous injection. 3 weeks after tumor inoculation, a rapid growth phase ensued possibly representing establishment of a supportive tumor vasculature. This rapid growth phase was accelerated to a much greater degree in SCD mice. Consistent with the hypothesis that this accelerated growth phase is promoted by tumor angiogenesis, tumors from SCD mice showed more vascularity compared to WT mice. Products of hemolysis may trigger both deleterious and protective effects in SCD. For example, hemin may induce acute chest syndrome in SCD [25] but may be protective in other SCD-related complications [26]. For example, administration of exogenous hemin has been shown to further increase the cytoprotective enzyme, HO-1 [27]. Of relevance, human sickle cell blood has been shown to induce HO-1 activity [28]. In this study, hemin induced HO-1 in endothelial cells in vitro, and tumors from SCD mice showed increased HO-1 activity in vivo. Since overexpression of HO-1 has been shown to promote melanoma tumor growth [4], we tested the causal role of HO-1 in this model by inhibiting HO-1 activity using ZnPP. HO-1 inhibition not only blocked the accelerated tumor growth and increased tumor vascularity but also blocked the effect of hemin on endothelial sprouting from aortic rings as well as the effect of SCD on angiogenic responses to matrigel *in vivo*.

### Study limitations

There was no effect of ZnPP on tumor growth in WT mice suggesting to us that HO-1 is particularly relevant in this melanoma model when upregulated, as in SCD. HO-1 is expressed by B16-F10 melanoma cells as well as several other host cell types [29–31]. We hypothesize that HO1 activity is enhanced in SCD due to endothelial cell or leukocyte production in response to products of hemolysis, however we have not proven the relevant cell type for effects we are observing in this model.

Whether the hemin mechanism is operative *in vivo* in this tumor model will require additional study. Additional inhibitors of HO-1, as well as antiangiogenic interventions will also be helpful to confirm relevant mediators involved in these observed effects.

## Conclusions

In summary, SCD is associated with enhanced melanoma growth in a murine model that is mediated by enhanced HO-1 and angiogenesis. Therapies targeting angiogenesis may be of particular use in SCD patients with malignant tumors.
